# A Linear Technique for Artifacts Correction and Compensation in Phase Interferometric Angle of Arrival Estimation †

**DOI:** 10.3390/s22041427

**Published:** 2022-02-13

**Authors:** Antonello Florio, Gianfranco Avitabile, Giuseppe Coviello

**Affiliations:** Department of Electrical and Information Engineering, Polytechnic University of Bari, 70125 Bari, Italy; antonello.florio@poliba.it (A.F.); gianfranco.avitabile@poliba.it (G.A.)

**Keywords:** Angle-of-Arrival estimation, phased arrays, phase interferometry, localization, error correction

## Abstract

Radio localization and radio positioning are relevant research fields for many telecommunications technologies. Usually, the solutions proposed by the literature rely on adaptive techniques related to some parameters that can be extracted from the received signal in cooperative device tracking. In this paper, we explore the artifacts that may be introduced into Angle-of-Arrival estimation based on phase interferometry, and we introduce a simple technique to mitigate their impact. Details of the mathematical discussion are presented and the approach is experimentally validated. The experimental results are compared with raw data to demonstrate the effectiveness of the proposed technique.

## 1. Introduction

Localization and positioning systems are very popular topics in the scientific community, given their relevance to many telecommunication applications. Positioning is an enabling factor for many activities, such as supporting users’ everyday life through navigation assistance systems [1], emergency response facilitation, collision avoidance in autonomous driving, defense purposes, and multirobot coordination tasks [2]. An alternative field of application for localization systems is telecommunications [3]. An example of this application is represented by the Massive MIMO systems (mMIMO) [4]. In fact, thanks to localization, it is possible to implement flexible communication pairs able to optimize channel capacity and the overall user experience by focusing the antenna system radiation pattern on a given direction or having multiple simultaneous, adaptively generated, very narrow beams pointing in different directions [5]. Other examples of applications are asset tracking, or in general, target tracking, and navigation assistance [3].

Adaptive algorithms require a variable to which relate the process. In particular, if it is necessary to focus the antenna beam towards a given direction, the variable is usually this direction. Over the decades, scientists have developed many techniques to extract the location of a given device from the physical properties of the electromagnetic (e.m.) signal it sends. One possible solution is represented by the Time of Arrival (ToA) or on the Time Difference of Arrival (TDoA) estimation of the signal at the receiver side [6]. Thanks to this timing information, it is possible to localize a device in 2D or 3D (depending on the number of receivers) by triangulation procedures. The choice among these two mentioned techniques depends on the achievable level of synchronization between transmitter and receiver(s). In fact, ToA requires knowledge of the e.m. signal’s Time of Departure (ToD) from the transmitter, or in general, its Time of Flight (ToF), and this is not always possible to measure. On the other hand, TDoA asks for multiple synchronized receivers, but no synchronization is required with the transmitter. Phase of Arrival (PoA) position estimation relies on the phase estimation, with which a given signal is received (or the phase difference between multiple receivers) [7]. They are mainly employed in the RFID positioning [8,9]. However, it is necessary to know the phase of the transmitted signal. Furthermore, particular attention must be put on the spatial/angular periodicity of the phase, that is 2π-periodic with distances that are multiples of the signal wavelength λ.

In this paper, we focus on the Angle of Arrival (AoA) estimation-based positioning. We define the AoA as the angle subtended by the broadside direction of a receiving array of antennas and the direction from which the received signal comes. Like in the TDoA case, multiple receivers are necessary for the estimation of the AoA. In particular, for the majority part of the implementations, the AoA information is extracted through an array of antennas. In fact, it is possible to link the AoA information to the phase difference between the signals arriving at the elements of the antenna array. Let us consider the simplest case of a Uniform Linear Array (ULA) of antennas with a spacing *d* and designed to operate at the central frequency *f*, whose associated wavelength in air is λ. Then, under the assumption of being in the far-field region, the phase difference between two array elements {ij}$\Delta \varphi_{ij}$ due to the AoA ϑ is

(1) $$\Delta \varphi_{ij}\left(\vartheta \right)=\frac{2\pi }{\lambda }\;\left(i-j\right)\;d\;\sin\vartheta$$

In order to estimate the AoA, one of the simplest techniques is the phase interferometric approach [10], which is particularly interesting for full-hardware AoA estimation systems such as the one proposed in [11]. The technique consists of simply reducing the AoA estimation to the cascade of a phase difference estimation, followed by the inversion of the phase–AoA relationship. Hence, in the ULA case, starting from (1), the estimated AoA due to the measured phase shift between antennas couple {ij}${\tilde{\vartheta }}_{ij}$ is:

(2) $${\tilde{\vartheta }}_{ij}=\arcsin(\frac{\lambda \;\Delta \varphi_{ij}\left(\vartheta \right)}{2\pi \;d\;(i-j)})$$

When dealing with this inversion, even small errors in the phase estimation step can corrupt the precision of the AoA value. In fact, let us suppose that a small phase estimation error δ is added to the real value (2). The AoA ϑ estimation ${\hat{\vartheta }}_{ij}$ becomes

(3) $${\hat{\vartheta }}_{ij}=\arcsin(\frac{\lambda \;(\Delta \varphi_{ij}\left(\vartheta \right)+\delta_{ij})}{2\pi \;d\;(i-j)})$$

since d=ψλ, ψ<1 for minimizing side-lobe effects [12], and δ≪2π, given that the expected phase errors are relatively small, we can rewrite (3) as

(4) $${\hat{\vartheta }}_{ij}=\arcsin(\frac{\Delta \varphi_{ij}\left(\vartheta \right)}{2\pi \;\psi \;(i-j)})+\arcsin(\frac{\delta_{ij}}{2\pi \;\psi \;(i-j)})={\tilde{\vartheta }}_{ij}+err_{ij}\left(\vartheta \right)$$

The aim of this paper is to propose a linear technique to minimize the term $err_{ij}\left(\vartheta \right)$ in order to correct the estimation to be as close as possible to the optimal value, which is represented by the theoretical AoA values. As will be explained in Section 2, there are many contributions to the phase error generation. The technique embeds in one single complex-valued matrix, the α-matrix, the amplitude, and the phase of the isofrequencial signals to be employed for the compensation of those effects.

### Contributions and Paper Organization

The main contributions offered by this study are here summarized:

- We mathematically introduce and experimentally prove, with a measurement campaign, the effectiveness of an artifacts linear compensation technique that can be employed when adopting the phase interferometric approach to AoA estimation.
- The technique embeds in one single computation all the possible mismatches due to systematic errors and a first-order (linear) approximation of the mutual coupling effects acting on the antenna array that can damage the integrity of the phase information.
- The matrix can be computed once and its values remain valid for the employed hardware and setup. Moreover, given the linearity of the approach, this is simple and fast to be implemented.
- Given the generality of the assumptions and the description, the technique can be implemented either in digital form by complex-number signal processing, or in the analog domain by means of Variable Gain Amplifiers (VGA) and networks of phase shifters.

The rest of the paper is organized as follows. In Section 2 we present in detail the analysis of the possible sources of error in an AoA estimation system based on phase interferometric approach. Later on, in Section 3, we present the theory behind the proposed error compensation technique. In Section 4, we present the experimental results of two AoA measurement campaigns conducted in an indoor environment. In particular, we first present the AoA estimation results in absence of the compensation technique, and then what we obtained by applying the technique. Furthermore, an experimental evaluation of the coherence time of the computed coefficients and on the compensation repeatability is furnished. Hence, conclusions close this work.

## 2. Problem Description

In this section, we discuss the possible sources of error when considering an AoA estimation system based on a phase interferometric approach. We aim to keep our discussion as general as possible, so we only assume that a separate RF front end is connected to any of the antennas composing the array used for the AoA estimation.

**Figure 1:**
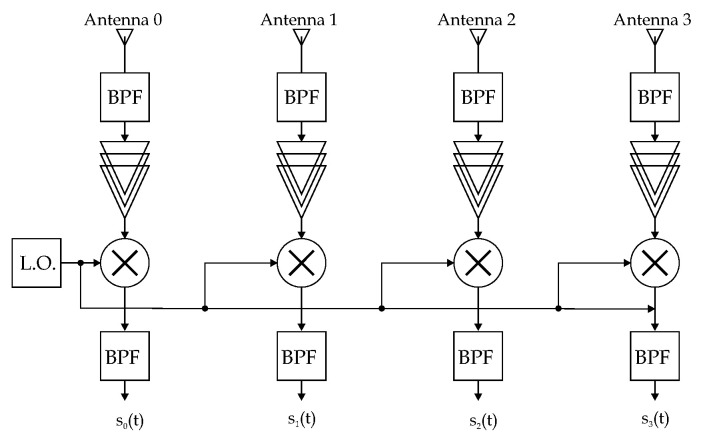
Block diagram depicting a 4-element ULA connected to a 4-channel RF front end with its basic sub-blocks for the amplification, down-conversion and band-pass filtering of the received signal, prior to the AoA estimation.

The RF front end is in charge of amplifying, down-converting, and filtering the received signal. A block diagram is proposed in Figure 1. We suppose every discrete component of the RF front ends (i.e., amplifiers, mixers, filters) to be nominally identical to the others. This choice is made because depending on the component quality and technology, the experimental results, and thus the sources and entity of artifacts, can be very different from the others. However, we focus our attention on two inevitable issues: the interconnection of those discrete elements and the mutual coupling of antenna elements in the array. For the first problem, we will consider the systematic error generated by signal paths’ length mismatches, and for the second issue, we will analyze the source of error coming from the mutual coupling of array elements. Strictly speaking, the latter is not a systematic error by its very definition, but we will approximate it as a systematic one, capturing what happens in a given position and trying to extend it to the others.

### 2.1. Phase Errors Due to Different Length of Signal Paths

As mentioned before, the different cable lengths and/or signal paths on the PCB interconnections introduce an error. In fact, it is important to remark that at higher frequencies, even small length mismatches impact on the phase of the traveling signals. Those length mismatches can occur for design errors, realization process tolerances, or in the case of cabling, by simply not choosing cables of the same length. Let us imagine two identical lines of length *l*, in which a signal of frequency *f* travels. By considering the two realizations of the lines $l_{1}$ and $l_{2}$ we see how $l_{2}=l_{1}+\Delta l$, hence, the line $l_{2}$ introduces a delay in the propagation of the signal. The introduced time delay is

(5) $$\tau \approx \frac{\Delta l}{c}\sqrt{\epsilon }$$

with ϵ the effective dielectric constant of the propagating medium. Remembering that a time delay τ corresponds to a phase shift of 2πfτ, we obtain that the phase error introduced by the lengths mismatch $err_{\Delta l}$ is

(6) $$err_{\Delta l}\approx 2\pi f\frac{\Delta l}{c}\sqrt{\epsilon }$$

Therefore, a length mismatch introduces a systematic error on the phase estimation that is linearly increasing with the difference in length of the paths.

### 2.2. Phase Artifacts Due to the Mutual Coupling of the Antenna Array Elements

As already mentioned in the introduction, the setup of an AoA estimation system usually is based on an array of antennas. Microstrip patch antenna arrays are very popular in telecommunication systems, offering a good trade-off between fabrication costs and radiation efficiency. However, this implementation also brings some contributions to the estimation errors due to the mutual coupling between the array elements.

Let us consider an array of microstrip patch antennas with spacing *d*. For sake of simplicity, we can consider the case of an ULA. According to what is described in [12], even choosing different alignments for the array elements leads to different mutual coupling decay speeds. In fact, a horizontal arrangement leads to higher decays than the vertical one.

It is well-known in the literature that mutual coupling is due to the near fields that exist along the air–dielectric interface [13]. If we call ρ the radial coordinate of the radiated field from the equivalent current element *J*, there are four possible field contributions [12]:

- Space waves, with $\rho^{-1}$ asymptotic radial decay;
- High-order waves, with $\rho^{-2}$ decay;
- Surface waves, with $\rho^{-0.5}$ decay;
- Leaky waves, with $e^{-\lambda \rho }\cdot \rho^{-0.5}$ decay;

From the infinite and infinitesimals analysis, on small distances (ρ→0, i.e., ρ≪λ), the surface and leaky waves are dominated by space and higher-order waves. Hence, the two former dominate on larger spacing, i.e., on large-size arrays their contribution cannot be supposed to be negligible.

Surface waves always exist and have 0 cut-off frequency [14]. However, their strength depends on the substrate thickness [13]. The surface wave couples with the feeding TEM mode as soon as the frequency increases. The lowest mode that can be coupled is the ${\mathrm{TM}}_{0}$, then the ${\mathrm{TE}}_{1}$, ${\mathrm{TM}}_{2}$, ... surface modes [13]. The cut-off frequencies are [13]:

(7) $$f_{c}\left(n\right)=\frac{nc}{4H\sqrt{\varepsilon_{r}-1}},\;\;n=0,1,2,\cdots$$

for a substrate with thickness *H* and relative dielectric constant $\varepsilon_{r}$. For thin substrate, the contribution is negligible. However, since thicker and low-density substrates are usually chosen to increase the bandwidth and the gain of a microstrip antenna [12], the surface wave contribution may be significant.

## 3. Theory of the α-Matrix

In order to simplify our model, let us consider a cluster of three antennas for the compensation procedure. The hypothesis does not impact the compensation effects on systematic errors, such as the path length mismatches. For what concerns antenna mutual coupling, we saw in the previous section that all the antenna elements in the array mutually interact. However, this interaction has a rapid decay. That given, our hypothesis for simplification is well-posed.

From a general point of view, two types of ports are present, the electrical and the radiative ones. The electrical ports are the physically accessible ports of the system (i.e., the ones from which the signal can be connected to a signal processor or a measurement unit). For what concerns the radiative ports, we can define them as the equivalent ports in the radiative plane, i.e., the ports on which the incident waves arrive when the ULA is in receiving mode. For an *N*-elements ULA, we can identify *N* radiative ports and *N* electrical ports.

For sake of simplicity, let us suppose a CW signal impinging on the array as a plane wave of frequency *f*. The equivalent voltage signal we measure at each electrical port of impedance $Z_{0}$ is represented by the phasor $\tilde{V_{i}},\;i=1,2,3$. In particular,

(8) $$\tilde{V_{i}}=\left|\tilde{V_{i}}\right|\;e^{j2\pi ft}\;e^{j∠\tilde{V_{i}}}\in C$$

Analogously, the incident power wave to the radiative port *i* has an equivalent voltage represented by the phasor $V_{i}$.

We describe the mutual coupling phenomena by means of a function $c_{ij}\left(\cdot \right)\in C$ that represents the coupling function associating the coupling source *j* and the coupled element *i*, modeling the coupling between radiative and electrical ports. In particular, it is simple to understand how the voltage signal at the *i*-th electrical port can be approximated as the composition of the contributions coming from the *i*-th antenna element and all the other contributions due to the coupling with the other two antennas, so that:

(9) $$\tilde{V_{i}}=\sum_{j=1}^{3}c_{ij}\left(V_{j}\right),\;\;\;j=1,2,3$$

Let us define the coupling function to be a linear complex-valued map $c_{ij}:C\to C$ such that

(10) $$c_{ij}:x\mapsto \alpha_{ij}x,\;\;\alpha_{ij}\in C$$

Hence, we can rewrite (9) as

(11) $$\tilde{V_{i}}=\sum_{j=1}^{3}\alpha_{ij}V_{j},\;\;\;j=1,2,3$$

or in matrix form,

(12) $$\left[\begin{array}{c} \tilde{V_{1}}\\ \tilde{V_{2}}\\ \tilde{V_{3}} \end{array}]=[\begin{array}{ccc} \alpha_{11} & \alpha_{12} & \alpha_{13}\\ \alpha_{21} & \alpha_{22} & \alpha_{23}\\ \alpha_{31} & \alpha_{32} & \alpha_{33} \end{array}]\;\;[\begin{array}{c} V_{1}\\ V_{2}\\ V_{3} \end{array}\right]$$

**Figure 2:**
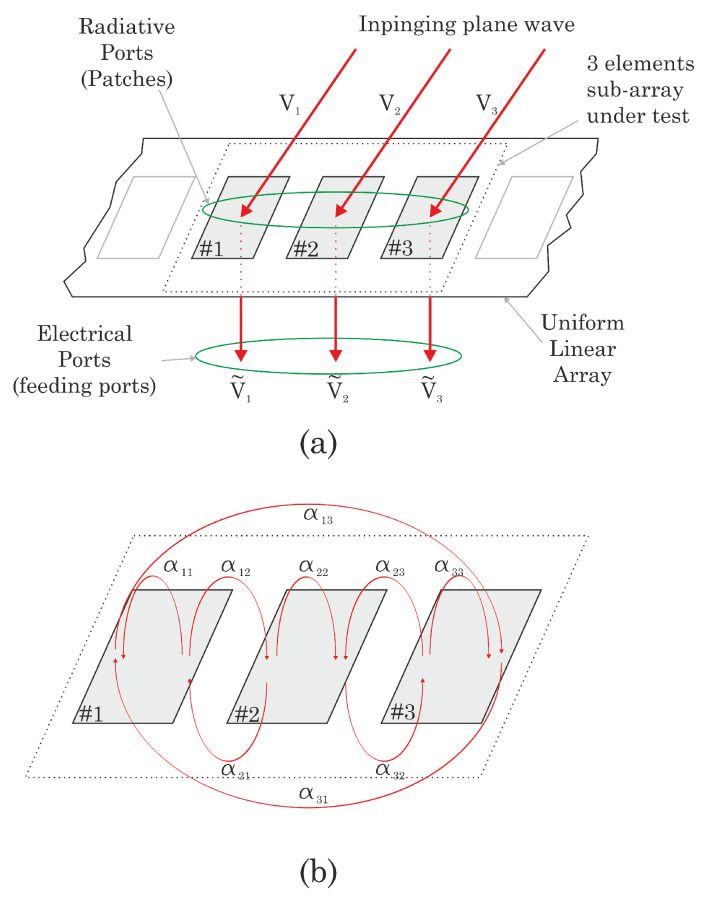
The 3-element subarray under test (**a**) and the interaction between the antenna array elements considered in the model (**b**).

The interaction between the array elements by means of the α−values is shown in Figure 2. The directions of the arrows evidence the role of the antennas in the coupling, that is, the pointer is the source of the coupling (*j*) and the pointee is the subject of the coupling (*i*). Ideally, in absence of coupling

(13) $$\left(\alpha_{ij}=1\Leftrightarrow i=j\right)\wedge \left(\alpha_{ij}=0\;\;\mathrm{elsewhere}\right)$$

so we can fix in our model $\alpha_{ii}=1$. Note that this is also true when amplifiers or attenuations are present in the chain, since we are supposing those to be equal for each channel.

For symmetry reasons which are evident in Figure 2, we can further simplify the model. In particular, if we hypothesize a symmetry with respect to the broadside axis, we can state that

(14) $$\alpha_{12}=\alpha_{32}$$

Therefore, by writing the relationships in matrix form, it is possible to obtain:

(15) $$\left[\begin{array}{c} \tilde{V_{1}}\\ \tilde{V_{2}}\\ \tilde{V_{3}} \end{array}]=[\begin{array}{ccc} 1 & \alpha_{23} & \alpha_{13}\\ \alpha_{23} & 1 & \alpha_{23}\\ -\alpha_{13} & -\alpha_{23} & 1 \end{array}]\;\;[\begin{array}{c} V_{1}\\ V_{2}\\ V_{3} \end{array}\right]$$

which is equivalent to writing it in a more compact form

(16) $$\tilde{V}=\alpha V$$

Note that with this formulation we also included all the possible systematic errors that are present and embedded in the ${\tilde{V}}_{i}$.

We aim to find the matrix α in order to recover the uncoupled voltages on the radiative ports V. However, we now have three equations and four unknowns, thus leading to $\infty^{1}$ possible solutions. In order to find a new constraint, we remember that an incoming signal from the broadside direction ϑ makes the receiving elements on the array experience different phase shifts according to their position. Let us take the central antenna i=2 as the reference. Hence, recalling (1),

(17) $$V_{i}=V_{2}\;\exp\left\{j\Delta \varphi_{i2}\left(\vartheta \right)\right\}\;\;\;i=1,2,3$$

the system (16) now becomes

(18) $$\tilde{V}=V_{2}\;\alpha \;\Phi \left(\vartheta \right)$$

being

(19) $$\Phi \left(\vartheta \right)=\left[\begin{array}{c} \exp\{j\Delta \varphi_{12}\left(\vartheta \right)\}\\ 1\\ \exp\{j\Delta \varphi_{32}\left(\vartheta \right)\} \end{array}\right]$$

By performing a calibration phase, it is possible to determine the α-matrix. In fact, if for example, we consider the broadside direction ϑ=0,

(20) $$\vartheta =0\Rightarrow \Delta \varphi_{i2}\left(\vartheta \right)=0,\;\;i=1,2,3$$

As a consequence, we evaluate the $\alpha_{ij}$ values by the ratio between the ${\tilde{V}}_{i}$.

Once the α-matrix has been computed, for each broadside position ϑ, the compensation is obtained by:

(21) $$V=\alpha^{-1}\tilde{V}$$

By its definition, the α-matrix is valid around a small enough interval of frequency, thus being valid for narrow-band signals. Moreover, it is possible to prove that, supposing an ideal downconversion stage, the α-matrix computed at the Intermediate Frequency (IF) is equal to the one that could have been computed at RF, with the great advantage of relaxing the complexity of the measurement hardware (the proof is in the Appendix A).

Note that along with the mutual coupling compensation, we are also unbiasing the measurement from the systematic errors by imposing the relationship (20).

## 4. Experimental Results

In this section, we prove the effectiveness of the proposed approach with an experimental measurement campaign, by analyzing the results and applying the proposed technique to the output data. The section organization is as follows. First, we describe the experiment setup, which will be the same for all the successive discussions. Then, we analyze the estimated AoAs without applying any compensation. After that, we calculate the α-matrix and analyze the time consistency of the computed values. Then, we will consider the effective compensation introduced by the α-matrix and compare it to the pure estimated AoA values in terms of absolute error with respect to the Ground Truth (GT) AoA values.

### 4.1. Experiment Setup

The experiment was set up in an indoor environment assuming a free Line-of-Sight (LOS) connection. The operating frequency was chosen to be $f_{\mathrm{RF}}=3.30$ GHz to prevent interferences from other telecommunications systems as Wi-Fi, Bluetooth, and so on. The transmitter was operated with a $f_{\mathrm{RF}}$ single-tone frequency and a −3 dBm power, using a microstrip antenna. On the receiving side, we set a four-element ULA of microstrip antennas with spacing d=λ/2, where λ is the open-air wavelength associated with $f_{\mathrm{RF}}$. Each element was connected to four nominally identical RF front-ends to amplify, down-convert and filter out the received signal to $f_{\mathrm{IF}}=210$ MHz. We employed an ADL5611 for the amplification stage and a custom-designed 5th order Chebyshev band-pass filter with center frequency 150 MHz and 80 MHz bandwidth. The output signals were then sampled with a 12-bit digital oscilloscope with 1 GHz bandwidth and 5 GS/s sample rate. The samples were then acquired on a 0.5 μs time window and processed with a custom MATLAB script.

The transmitter was placed at broadside distances $l_{k}=\left\{2.10,3\right\}$ m, both of them greater than the Fraunhofer distance, allowing the wave to be considered plane. Then, each measurement was taken by translating in the direction parallel to the array, starting from the array center position, taken as reference. The relative position for the distance *l* with respect to the array center position for experiment *j* is $x_{j}$. For each $x_{j}$ we acquired 50 snapshots (one every 2 s). The GT angles were computed as

(22) $$\mathrm{GT}\left(x_{k}\right)=\arctan\left(\frac{x_{k}}{l_{k}}\right)\;\frac{180}{\pi }$$

In Table 1, we summarized the mean value of the signal-to-noise ratio (SNR) of all the positions for each experiment.

### 4.2. AoA Estimation without Compensation

The AoA estimation was conducted by employing the already-introduced phase interferometric approach. The phase difference values were extracted through the algorithm described in [15]. For an *N*-element array, we achieved, in far-field, up to *Q* AoA estimations for the same GT angle, by taking the distinct Q couples of antennas and properly computing the phase difference, with Q

(23) $$Q=\sum_{j=1}^{N}\left(N-j\right)$$

**Figure 3:**
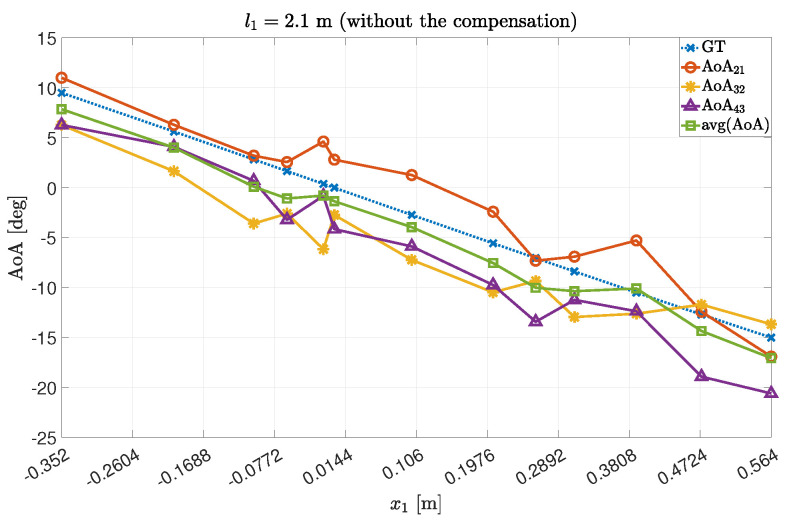
AoA data concerning Exp.#1. In detail, the AoA were computed thanks to the couples {21},{32},{43} and their average.

**Figure 4:**
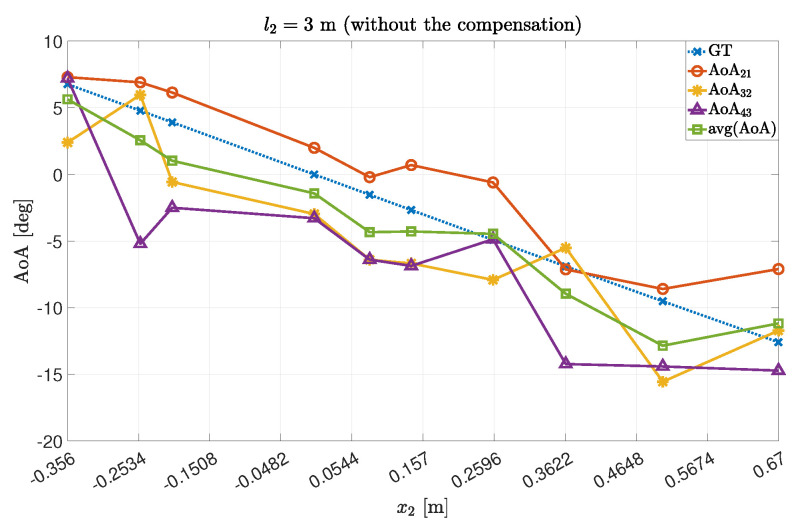
AoA data concerning Exp.#2. In detail, the AoA were computed thanks to the couples {21},{32},{43} and their average.

Accordingly, we obtained the following AoA values by averaging on a couple-by-couple basis the estimated AoAs, to improve the precision. The results are summarized in Figure 3 and Figure 4 for the experiments 1 and 2, respectively.

The obtained values were compared to the theoretical ones (the Ground Truth angles, GT).

In Table 2, we summarized the statistics regarding the computed absolute errors. In particular, the absolute error on the AoA estimated with the antenna array couple {ij} during the experiment *k* for the broadside position $x_{k}$ is computed as

(24) $$err_{\left\{ij\right\}}^{k}=\left|\mathrm{GT}\left(x_{k}\right)-{\mathrm{AoA}}_{\left\{ij\right\}}\left(x_{k}\right)\right|$$

As expected, we obtained better results by averaging the values, in terms of both average and error dispersion. However, the results are still biased by the presence of non-negligible error components.

### 4.3. Computation and Analysis of the α-Matrix

As described in Section 3, the α-matrix was computed starting from the measured values when the transmitter is in the array broadside center position. Since the method is defined for three contiguous antennas, we chose to apply it to the subarray {2,3,4}. The α-matrix computation is demanded to a MATLAB script.

**Figure 5:**
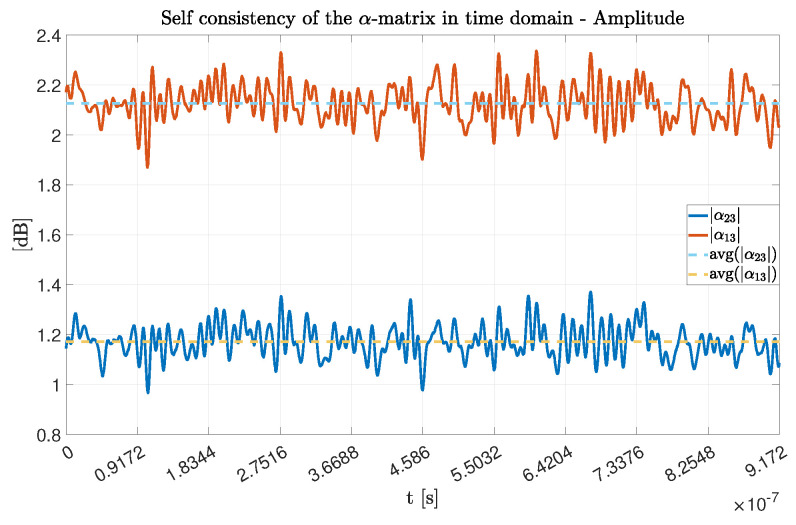
Amplitude values for the α-matrix coefficient during an experimental snapshot.

We assumed the α-matrix to be computed once for all the successive measurements, with a unique value. We want to prove here this assumption. To do that, first, we studied the behavior of its coefficients in the time domain, during the acquisition time of one experimental sample. We took the first acquisition of Exp#1 as the reference. Concerning the amplitude, it is normally distributed around the mean value, as shown in Figure 5.

**Figure 6:**
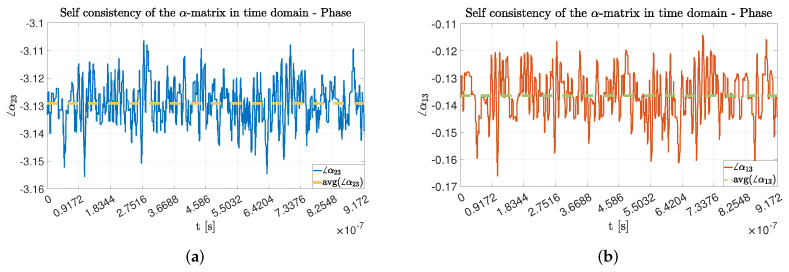
Phase values for the α-matrix coefficient during an experimental snapshot. In particular, in (**a**) phase values for the $\alpha_{23}$ coefficient and in (**b**) phase values for the $\alpha_{13}$ coefficient.

In particular, as described in Table 3, the standard deviation value being an order of magnitude lower than that of the mean value, we can assume the mean value to be a good approximation of the dynamic behavior. Furthermore, in terms of phase values, as seen in Figure 6 they expose the same statistical properties. We can then conclude that the computed mean values for the α-matrix amplitude and phases are a good approximation of the overall dynamic behavior during the acquisition time window.

**Figure 7:**
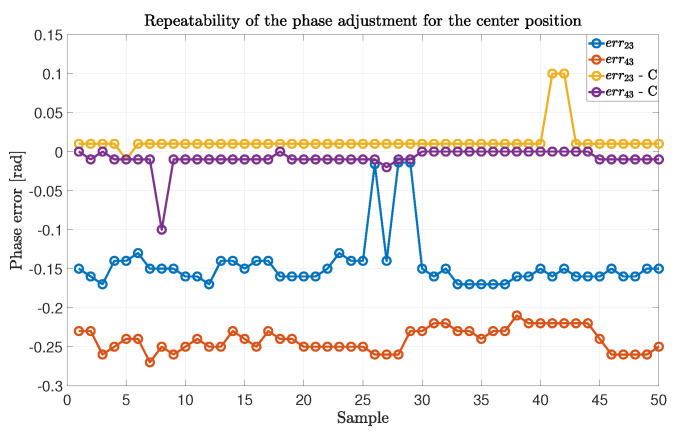
Analysis of the repeatability of the phase adjustment for the center position on 50 experimental samples, with $err_{ij}$ representing the absolute error committed for the antenna elements {ij} without compensation and $err_{ij}$-C the absolute error committed with the compensation procedure.

We now prove that the α-matrix computation is valid for the successive measurements. In other words, we show how the compensation introduced by the matrix is usable for the successive samples after the calibration phase. To do that, we took the compensation errors for the center position for each sample and for each antenna couple {23},{43}. The results are shown in Figure 7, where it is possible to see how the phase errors for the uncompensated values are not stable, while the compensated values are stable for the entire duration of the experiment, except for a few outliers.

### 4.4. AoA Estimation with Compensation

In this section, we analyze what we obtained using the α-matrix compensation on the experimental samples. In particular, once the matrix was computed for the center position, it was stored and recalled for the computation of the compensated waveforms for each position. The compensated waveforms were then employed for the AoA estimation, as previously described. The validation of this approach was performed with a custom MATLAB script.

**Figure 8:**
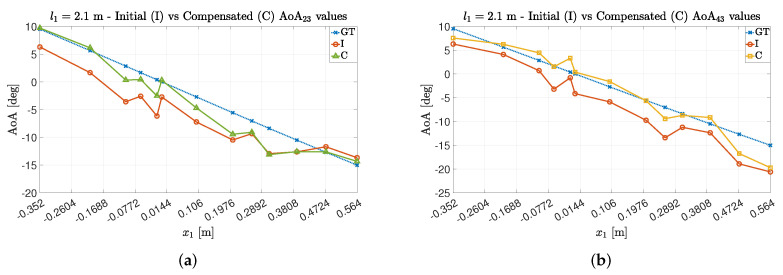
AoA estimation values for Exp#1 before and after compensation obtained for (**a**) the antenna couple {23} and (**b**) the antenna couple {43}.

**Figure 9:**
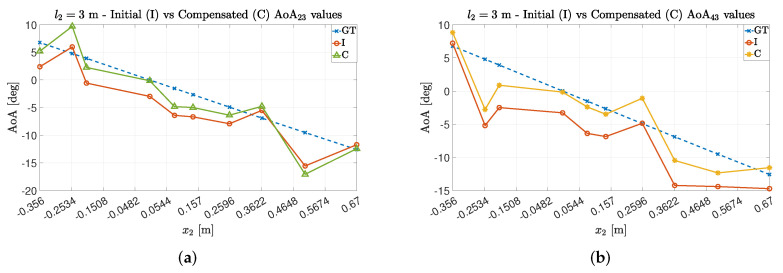
AoA estimation values for Exp#2 before and after compensation obtained for (**a**) the antenna couple {23} and (**b**) the antenna couple {43}.

For both experimental trials Exp#1 and Exp#2, it is possible to see (in Figure 8 and Figure 9) how the compensation method allowed the estimation points to be near the expected ones. In particular, for Exp#2, the impact of the multipath phenomenon on the estimation is clear, since greater distances between the transmitter and receiver allow for both decreasing the power associated with the LoS component and relatively increasing the impact of the other paths on the estimation value [16]. However, even in this case, the estimation quality is improved by the adoption of the proposed technique.

**Figure 10:**
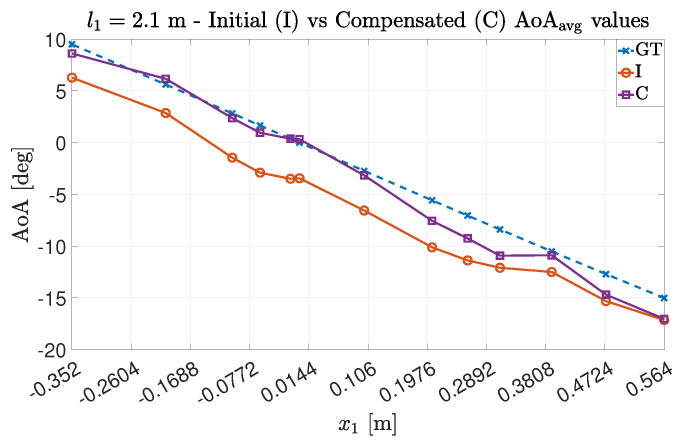
Average AoA estimation values for Exp#1 before and after the compensation.

**Figure 11:**
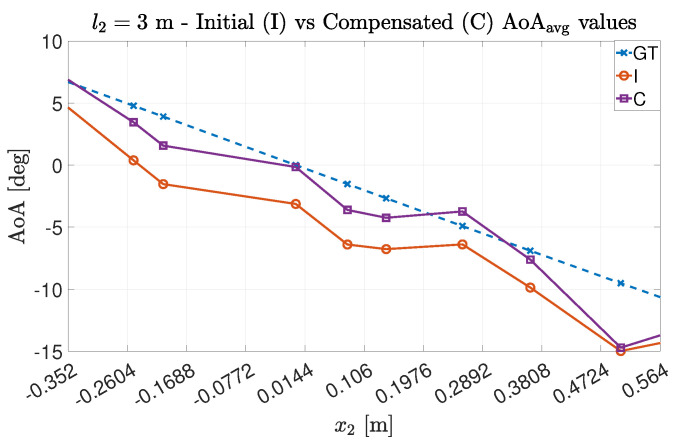
Average AoA estimation values for Exp#2 before and after the compensation.

As it was conducted in the uncompensated case, we studied the impact of an averaging operation between the estimated AoA values. The results are shown in Figure 10 for Exp#1 and in Figure 11 for Exp#2. As expected, the estimation quality improves even more: in Exp#1, the estimated values are very close to the GT ones, while for Exp#2, the introduction of the averaging better mitigates the estimation artifacts.

### 4.5. Comparison

We now compare the uncompensated and compensated AoA estimations. In order to properly compare them, we employ the absolute error metric (24) introduced in Section 4.2. We start by comparing the estimations made by antenna couples {23} and {43}. The results and the percentage comparison with respect to the absolute errors committed without the compensation procedure (see Table 2) are shown in Table 4. Table 5 shows the AoA estimation errors obtained after averaging the estimated AoAs after the compensation for the same antenna couples as before.

**Figure 12:**
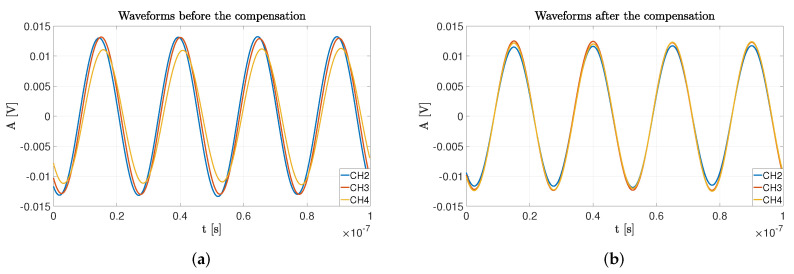
Time- domain comparison of the (**a**) acquired waveforms versus the (**b**) compensated waveforms in the Exp #2 calibration point. The initial waveforms were preprocessed through a digital FIR filtering and downconversion stage to the IF of 40 MHz for better view the results.

It is simple to see how the average absolute errors decrease by more than one half in the Exp#1 case and more than one-fourth in the Exp#2 case. Additionally, standard deviations experience a reduction in their value. For better understanding the compensation action, in Figure 12 it is evident how the initial artifacts in the calibration points are almost canceled by the compensation procedure.

If we consider the estimation made by averaging the AoA values after having been compensated, we see how, even in this case, a good reduction in the average absolute error is obtained. However, in this case, the compensation introduces a distortion of the distribution of the errors around the mean value, even if it is not so strong in absolute terms.

## 5. Conclusions and Future Work

We introduced the α-matrix, a linear technique for artifact correction and compensation in phase-interferometric AoA estimation for radio localization. A mathematical discussion was conducted in detail. This technique embeds in one single computation all the possible mismatches due to systematic errors and first-order (linear) approximation of the mutual coupling effects acting on the antenna array. There is great advantage in the α-matrix being computed once forever in the calibration phase. Additionally, given the generality of the description, the technique is suitable for being implemented either in the digital or analog domain.

The experimental campaign validated the quality of the compensation introduced by halving the error trend even in the indoor environment, where multipath has a strong influence on the AoA estimation error.

We remark that by its definition, the α-matrix is valid around a small enough interval of frequencies around the central one.

Future work can be conducted on extending the proposed approach to *N*-array elements with different arrangements for the subarray employed in the compensation and on cascading established, and efficient real-time AoA estimation algorithms to this approach in order to validate the minimal timing impact introduced by the technique.

## Figures and Tables

**Table 1:** Average SNR values for channels and experiments.

Exp#	Average SNR [dB]				
	**CH1**	**CH2**	**CH3**	**CH4**	**AVG**
1	29.55	30.61	28.56	29.54	29.56
2	28.87	31.20	27.26	28.17	28.88

**Table 2:** Statistic indexes on the AoA estimation made without calibration.

	${\mathrm{err}}_{21}^{k}$ [deg]		${\mathrm{err}}_{32}^{k}$ [deg]		${\mathrm{err}}_{43}^{k}$ [deg]		${\mathrm{err}}_{\mathrm{avg}}^{k}$ [deg]	
	**avg**	**std**	**avg**	**std**	**avg**	**std**	**avg**	**std**
Exp#1	2.06	1.67	3.68	1.76	3.64	1.75	1.81	0.72
Exp#2	2.25	1.69	3.32	1.74	4.36	3.07	1.93	0.89

**Table 3:** Statistical indexes of the amplitude and phase of the α-matrix coefficients on the 50 snapshots of the single experimental calibration point.

	avg(|·|) [dB]	std(|·|) [dB]	avg(∠·) [rad]	std(∠·) [rad]
$\alpha_{23}$	1.17	0.06	−3.13	0.007
$\alpha_{13}$	2.13	0.07	−0.13	0.008

**Table 4:** Statistical indexes of the absolute AoA estimation errors after compensation for antenna couples {23} and {43}.

	${\mathrm{err}}_{23}^{k}$ [deg]				${\mathrm{err}}_{43}^{k}$ [deg]			
	**avg**		**std**		**avg**		**std**	
Exp#1	1.78	−51.6%	1.47	−16.4%	1.66	−54.4%	1.49	−14.9%
Exp#2	2.51	−24.4%	2.25	−22.7%	2.57	−41.0%	2.16	−29.6%

**Table 5:** Statistical indexes of the absolute AoA estimation errors after averaging the estimated AoAs after compensation for antenna couples {23} and {43}.

	${\mathrm{err}}_{\mathrm{avg}}^{k}$ [deg]			
	**avg**		**std**	
Exp#1	1.11	−38.7%	0.88	+18.1%
Exp#2	1.54	−54.0%	1.47	+39.4%

## Appendix A. RF/IF α-Matrix Equivalence

**Theorem** **A1.**
*Let $\alpha (f_{\mathrm{RF}})$ the α-matrix computed at frequency $f_{\mathrm{RF}}$. Supposing an ideal downconversion stage, its values are proportional to the ones computed at IF $f_{\mathrm{IF}}$, that is*

(A1)
$$\alpha \left(f_{\mathrm{IF}}\right)=\dot{K}\;\alpha \left(f_{\mathrm{RF}}\right),\;\;\dot{K}\in C$$

**Proof.** Starting from (16) we can write

(A2) $$\alpha \left(f_{\mathrm{RF}}\right)={\tilde{V}}_{\mathrm{RF}}V_{\mathrm{RF}}^{-1}$$

with ${\tilde{V}}_{\mathrm{RF}},V_{\mathrm{RF}}$ being the same matrices as before, with the subscript denoting their relationship with RF frequency, so prior to the downconversion. Moreover, we can also write

(A3) $$\alpha \left(f_{\mathrm{IF}}\right)={\tilde{V}}_{\mathrm{IF}}V_{\mathrm{RF}}^{-1}$$

since the downconversion stage is operated only on the signals coming from the electrical ports. In order to prove the statement, we should find the phasor $\dot{K}\in C$ such that

(A4) $${\tilde{V}}_{\mathrm{RF}}=\dot{K}\;{\tilde{V}}_{\mathrm{IF}}$$

However, denoting the downconversion to frequency $f_{\mathrm{IF}}$ as the linear operator ⊗ such that

(A5) $$⊗\left(s\left(t\right),f_{\mathrm{IF}}\right)=\mathrm{LPF}\left\{s\left(t\right)\cdot \exp\left[j2\pi \;(f_{\mathrm{RF}}-f_{\mathrm{IF}})t\right]\right\}$$

with LPF{·} being a low-pass filtering operation performed by a Linear Time Invariant (LTI) filter, we can see that

(A6) $${\tilde{V}}_{\mathrm{IF}}=⊗\left({\tilde{V}}_{\mathrm{RF}},f_{\mathrm{IF}}\right)$$

hence,

(A7) $$\alpha \left(f_{\mathrm{IF}}\right)=⊗\left({\tilde{V}}_{\mathrm{RF}},f_{\mathrm{IF}}\right)V_{\mathrm{RF}}^{-1}$$

and since ⊗(·) was defined as a linear operator over complex values, we can write

(A8) $$\alpha \left(f_{\mathrm{IF}}\right)=\dot{K}\;{\tilde{V}}_{\mathrm{RF}}V_{\mathrm{RF}}^{-1}=\dot{K}\alpha \left(f_{\mathrm{RF}}\right)$$

□

## References

[1] Bounini F., Gingras D., Pollart H., Gruyer D. (2021). From Simultaneous Localization and Mapping to Collaborative Localization for Intelligent Vehicles. IEEE Intell. Transp. Syst. Mag..

[2] Zekavat S.A.R., Kansal S., Levesque A.H. (2011). Wireless Positioning Systems: Operation, Application, and Comparison. Handbook of Position Location.

[3] Zekavat S., Buehrer R., Durgin G., Lovisolo L., Wang Z., Goh S.T., Ghasemi A. (2021). An Overview on Position Location: Past, Present, Future. Int. J. Wirel. Inf. Netw..

[4] Björnson E., Sanguinetti L., Wymeersch H., Hoydis J., Marzetta T.L. (2019). Massive MIMO is a reality—What is next?: Five promising research directions for antenna arrays. Digit. Signal Process..

[5] Larsson E.G., Edfors O., Tufvesson F., Marzetta T.L. (2014). Massive MIMO for next generation wireless systems. IEEE Commun. Mag..

[6] Piccinni G., Avitabile G., Coviello G., Talarico C. (2020). Real-Time Distance Evaluation System for Wireless Localization. IEEE Trans. Circuits Syst. I Regul. Pap..

[7] Zafari F., Gkelias A., Leung K.K. (2019). A Survey of Indoor Localization Systems and Technologies. IEEE Commun. Surv. Tutor..

[8] Povalac A., Sebesta J. Phase of arrival ranging method for UHF RFID tags using instantaneous frequency measurement. Proceedings of the 2010 Conference Proceedings ICECom, 20th International Conference on Applied Electromagnetics and Communications.

[9] Scherhäufl M., Pichler M., Müller D., Ziroff A., Stelzer A. Phase-of-arrival-based localization of passive UHF RFID tags. Proceedings of the 2013 IEEE MTT-S International Microwave Symposium Digest (MTT).

[10] International Telecommunications Union (I.T.U.) Spectrum Monitoring Handbook. http://handle.itu.int/11.1002/pub/80399e8b-en.

[11] Avitabile G., Florio A., Coviello G. (2020). Angle of Arrival Estimation through a Full-Hardware Approach for Adaptive Beamforming. IEEE Trans. Circuits Syst. II Express Briefs.

[12] Balanis C.A. (2015). Antenna Theory: Analysis and Design.

[13] Pozar D. (1983). Considerations for millimeter wave printed antennas. IEEE Trans. Antennas Propag..

[14] Mailloux R.J. (2017). Phased Array Antenna Handbook.

[15] Florio A., Avitabile G., Coviello G. Digital Phase Estimation through an I/Q Approach for Angle of Arrival Full-Hardware Localization. Proceedings of the 2020 IEEE Asia Pacific Conference on Circuits and Systems (APCCAS).

[16] Florio A., Avitabile G., Coviello G., Ma J., Man K.L. The Impact of Coherent Signal Reception on Interferometric Angle of Arrival Estimation. Proceedings of the 2020 International SoC Design Conference (ISOCC).

